# Evaluating Melanoma Risk in Adult Mastocytosis: Potential Impact of Detection Bias – A Registry-based Study (Sweden)

**DOI:** 10.2340/actadv.v105.43052

**Published:** 2025-08-18

**Authors:** Anna BERGSTRÖM, Hans HÄGGLUND, Anders BERGLUND, Gunnar NILSSON, Mats LAMBE

**Affiliations:** 1 Department of Medical Sciences, Dermatology and Venereology, Uppsala University, Akademiska University Hospital, Uppsala; 2 Department of Medical Sciences, Hematology, Uppsala University, Uppsala; 3 Epistat, Uppsala; 4 Department of Medicine Solna, Division of Immunology and Allergy, and Center for Molecular Medicine, Karolinska Institutet and Karolinska University Hospital, Stockholm; 5 Department of Medical Sciences, Hematology, Uppsala University, Uppsala; 6 Department of Medical Epidemiology and Biostatistics, Karolinska Institutet, Stockholm, Sweden

**Keywords:** basal cell carcinoma, comorbidity, malignant melanoma, mastocytosis, melanoma *in situ*, Sweden

## Abstract

There is some evidence that mastocytosis patients are at increased risk of skin cancer. This study aimed to assess the risk of malignant melanoma (MM), melanoma *in situ (Mis)*, and basal cell carcinoma (BCC). A dataset was generated by individual-level record linkages between Swedish population registers including the National Patient Register (NPR), the Swedish Cancer Register (SCR), and the Population Register (PR). Adult patients with a mastocytosis diagnosis between 2001 and 2018 were identified in the SCR and NPR. For each case, 5 mastocytosis-free comparators matched on age, sex, and county of residence were randomly chosen from the PR. Records of skin cancer were identified in the SCR and NPR. In total, the study encompassed 2,040 mastocytosis patients of whom 63 had a record of MM/Mis and 168 a record of BCC. Compared with comparators, the risk of MM/Mis was more than twofold higher (OR 2.39, 95% CI 1.8–3.2). Risk estimates for BCC were also elevated (OR 1.77, 95% CI 1.49–2.14). When assessing the timing of skin cancers, a substantial portion were diagnosed near index date. Taken together, in the present study these findings of increased risk of MM/Mis and BCC in mastocytosis patients may reflect an influence of detection bias.

## SIGNIFICANCE

This study found that the risk of skin cancer was higher in individuals with mastocytosis compared with the background population represented by mastocytosis-free comparators. In the present study, we also found that a substantial proportion of skin cancer cases were detected near the date of mastocytosis diagnosis, pointing to a possible influence of detection bias. Skin checkups of mastocytosis patients should focus not only on mastocytosis-related skin changes, but also on surveillance for lesions with malignant potential.

Mast cells (MCs) are important for homeostasis and surveillance of the human system, recognizing both endogenous and exogenous agents (1). MCs are known for their role in allergic diseases, but they also contribute to other conditions (2). Mastocytosis is a condition characterized by focal accumulation of abnormal MCs in 1 or more tissues, with mediator-related symptoms (3). Depending on the spread of the abnormal MCs, the disease is divided into cutaneous mastocytosis, without systemic involvement, and systemic mastocytosis (SM). In SM the MCs form infiltrates in various internal organs, in contrast to cutaneous mastocytosis, where the MC infiltrate is restricted to the skin (3). Cutaneous involvement in patients with mastocytosis is heterogeneous and a classification for cutaneous mastocytosis has been proposed (4). Overall, more than 80% of all patients with mastocytosis, regardless of type of mastocytosis, exhibit characteristic brown or red skin lesions (5).

In a recent Swedish population-based study, we observed an annual incidence of adult mastocytosis of 1.56 per 100,000 and a prevalence of 23.9 per 100,000, estimates higher than previously reported (6). The median age of diagnosis was 50 years. In addition, we found evidence of a higher comorbidity burden in adult mastocytosis patients compared with the background population, including cancer, diabetes, chronic pulmonary disease, renal disease, and paraplegia and hemiplegia (6). An increased risk of anaphylaxis (7), osteoporosis (8,9), solid cancers, venous thromboembolism, and stroke (10) has previously been described. Also, a higher baseline prevalence of metastatic cancers, chronic pulmonary diseases, and connective tissue diseases has been reported (11). Specifically, several case reports have suggested that mastocytosis is associated with an increased risk of malignant melanoma (MM) (12–19), findings further supported in a Danish population-based study including 687 adult mastocytosis patients that reported increased risks of both MM (Hazard ratio (HR) 7.5) and non-melanoma skin cancer (NMSC) (HR 2.5) (10).

In recent decades, the incidence of MM has risen sharply in Western countries, including Sweden, with more than 5,000 cases reported annually with a median age of diagnosis of 65 years in women and 70 years in men. Although the increase can largely be attributed to changes in UV exposure, it is likely that an increased diagnostic intensity with earlier detection has also played a role. In Sweden, the increase of early stage, thin MMs has been especially pronounced (20). The number of incident cases of the most common type of NMSC, basal cell carcinomas (BCCs), is more than 10 times that of MM with a median age of diagnosis of around 70 years (21).

The aims of the present population-based study were twofold. First, to examine the risk of developing MM and/or melanoma *in situ* (Mis) in patients with mastocytosis compared with the background population. Second, to assess whether a history of MM and/or Mis is more common in individuals with mastocytosis. For comparison, we also assessed the risk of BCC, a skin tumor originating from other cell types, but with a similar median age at diagnosis.

## MATERIALS AND METHODS

### Study design and study population

A dataset was generated based on individual-level record linkages between 2 registers with national coverage, i.e., the Swedish Patient Register (NPR) and the Swedish Cancer Register (SCR) at the National Board of Health and Welfare. The NPR includes data based on the International Classification of Diseases (ICD) and the SCR contains detailed information coded according to the Systematized Nomenclature of Medical-Clinical Terms (SNOMED). ICD-10 and SNOMED codes used to identify a cohort of mastocytosis are shown in Table SI.

By use of information in the in- and outpatient NPR and the SCR we were able to identify patients with a record of a primary or secondary diagnosis of mastocytosis between 2001 and 2018. The primary diagnosis is defined as the main reason the patient is seeking care and the secondary diagnosis is any condition that exists alongside the primary diagnosis. Individual-level record linkages were made possible by use of the unique personal identity number assigned to all residents in Sweden at birth or on permanent residency. Thus, one individual could only be included in the study once, even though the diagnosis might have been recorded multiple times. Cases were included based on the first registered diagnosis. For information on baseline MM or Mis prevalence and the risk of developing MM or Mis in mastocytosis patients, we used information in the NPR and SCR. In this way, records of a MM or Mis diagnosis, both before and after the date of the mastocytosis diagnosis, could be identified.

In a separate step, we also assessed the risk of BCCs, which originate from different cells but share the major risk factor with MM/Mis (sun/UV exposure), and with a similar median age at diagnosis. BCCs were identified in the NPR, before and after the date of the mastocytosis diagnosis. ICD-10 codes used to identify MM, Mis, and BCC are shown in Table SI.

For each mastocytosis case, the date set also included 5 randomly selected comparators free of mastocytosis, identified in the Population Register (PR) and matched on sex, age, and county of residence. To assess mastocytosis status and comorbidity burden, comparators were cross-linked to the SCR and the NPR.

### Statistical analysis

Descriptive statistics were used to characterize mastocytosis patients and comparators. Categorical variables were presented as numbers and percentages. Statistical analyses were performed to assess the risk of developing MM or Mis in mastocytosis patients compared with mastocytosis-free comparators. We also compared the prevalence of MM and Mis at index date (date of mastocytosis diagnosis). The cumulative incidence of MM in mastocytosis patients was compared with the incidence in mastocytosis-free comparators. A 5% significance level was considered statistically significant. All statistical analyses were performed using R Statistical Software (v 4.1.2 R Core Team 2021; R Foundation for Statistical Computing, Vienna, Austria).

## RESULTS

We identified 2,040 individuals diagnosed with mastocytosis during adulthood (≥ 20 years) and a cohort of 10,193 mastocytosis-free comparators. The majority of the patients were identified in the NPR outpatient register. Some patients were registered in multiple registries, hence the total sum is larger than the final study population. The median age at diagnosis was 50.6 years, with the majority being diagnosed in the age group 40–49 years. Baseline characteristics of the mastocytosis cohort are presented in **Table I**. Number of patients and diagnostic codes by register sources are shown in Table SII.

**Table I:** Characteristics of the mastocytosis cohort

All subjects	*n* (%)
Total	2,040 (100)
Gender	
Female	1,211 (59.4)
Male	829 (40.6)
Age at diagnosis	
20–29 years	284 (13.9)
30–39 years	328 (16.1)
40–49 years	388 (19.0)
50–59 years	365 (17.9)
60–69 years	345 (16.9)
70–79 years	227 (11.1)
80–89 years	91 (4.5)
> 90 years	12 (0.6)
Data source	
NPR, outpatient	1,912
NPR, inpatient	330
SCR	175

NPR: National Patient Register; SCR: Swedish Cancer Register.

### Risk of malignant melanomas and melanoma *in situ* in individuals with mastocytosis

A total of 39 (1.9%) mastocytosis patients had a record of MM in the NPR or SCR before (*n* = 27) or after (*n* = 12) the mastocytosis diagnosis. Another 24 (1.2%) patients had a record of Mis, of whom 6 were diagnosed before and 18 after the index date. The corresponding numbers of MMs/Mis before and after index date in the mastocytosis-free comparators were 81 (0.8%) and 53 (0.5%). In total, the risk of MM and Mis was more than twice as high in individuals with mastocytosis (Odds ratio (OR) 2.43; 95% Confidence Interval (CI) 1.66–3.58 and OR 2.28; 95% CI 1.4–3.7, respectively) (**Table II**).

**Table II:** Risk of malignant melanoma (MM) and melanoma *in situ* (Mis) in mastocytosis patients compared with mastocytosis-free comparators

Item	No (%)	Yes (%)	Total	Diagnosis before index date
MM				
Cases	2,001 (98.1)	39 (1.9)	2,040	27
Comparators	10,112 (99.2)	81 (0.8)	10,193	44
OR (95% CI)	2.43 (1.66–3.58)			
Mis				
Cases	2,016 (98.8)	24 (1.2)	2,040	6
Comparators	10,140 (99.5)	53 (0.5)	10,193	24
OR (95% CI)	2.28 (1.4–3.70)			
MM + Mis (total)				
Cases	1,977 (96.9)	63 (3.1)	2,040	33
Comparators	10,059 (98.7)	134 (1.3)	10,193	68
OR (95% CI)	2.39 (1.8–3.2)			

OR: odds ratio; CI: confidence interval.

No = no MM/Mis. Yes = MM or Mis.

### Timing and risk of malignant melanoma and melanoma *in situ* diagnosis in relation to date of mastocytosis diagnosis

**Fig. 1:**
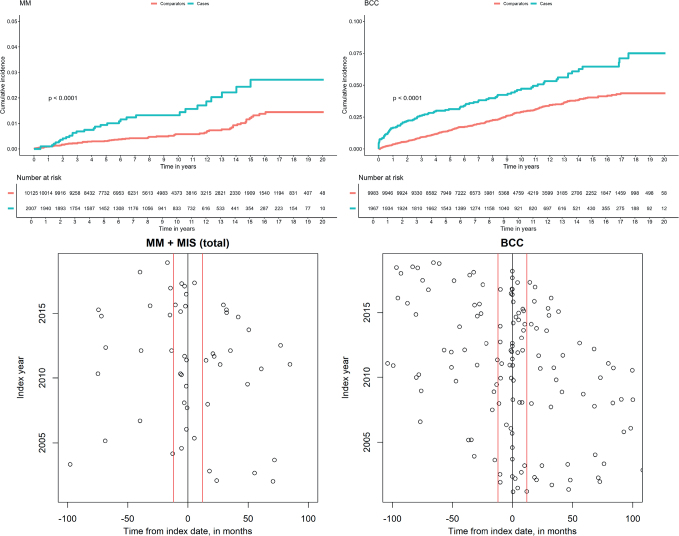
Cumulative incidence of MM/Mis and BCC and the timing of the skin cancer diagnosis in relation to mastocytosis diagnosis.

In a separate step, the timing and risk of MM and Mis in relation to the index date was assessed **(****Fig. 1**). 33 (52%) out of 63 patients had their diagnosis before index date, and 15 (45%) out of 33 patients had their diagnosis within 12 months prior to the index date (**Table III**, scenario 2). This number remained unchanged when this time window was extended to 3 years prior to index date (scenario 3). Thus, while 15 cases were diagnosed in the 12-month period, the remaining 18 of the 33 MM and Mis cases were diagnosed more than 3 years before the mastocytosis diagnosis. While the majority of mastocytosis patients with MM received their diagnosis before the index date (27/39), most individuals with Mis were recorded after the mastocytosis diagnosis (18/24) (Table II).

**Table III:** Risk of MM and Mis after mastocytosis diagnosis based on 3 scenarios: (1) after date of mastocytosis diagnosis, (2) including a 1-year period before mastocytosis diagnosis, (3) including a 3-year prior period

Item	Events	Person years	HR (95% CI)	*p*-values
Scenario 1				
Cases	30	50.4	2.39 (1.55–3.68)	< 0.001
Comparators	66	265.0	1.00 (ref)	
Scenario 2				
Cases	45	55.9	3.08 (2.13–4.56)	< 0.001
Comparators	76	292.7	1.00 (ref)	
Scenario 3				
Cases	45	55.9	3.08 (2.13–4.56)	< 0.001
Comparators	76	292.7	1.00 (ref)	

In Table III, estimates of the risk of MM and Mis after mastocytosis based on 3 scenarios are presented. In scenario 1, strictly based on events following the date of mastocytosis diagnosis, the magnitude of the elevated risk (HR 2.39; 95% CI 1.55–3.68) was similar to that presented in Table II for the risk of MM and Mis diagnosed before or after mastocytosis (OR 2.39; 95% CI 1.8–3.2). In scenario 2, the risk estimate also included MM and Mis diagnosed within 1 year before the date of mastocytosis diagnosis, yielding a risk estimate of HR 3.08 (95% CI 2.13–4.56). Numbers and risk estimates did not change when a 3-year prior to mastocytosis diagnosis was assessed (scenario 3).

### Risk of basal cell carcinoma in individuals with mastocytosis

A total of 168 (8.2%) mastocytosis patients had a record of BCC in the NPR before (*n* = 73) or after (*n* = 95) the mastocytosis diagnosis. The corresponding number of BCCs in the mastocytosis-free comparators were 487 (4.8%), yielding an OR of 1.79 (95% CI 1.49–2.14) (**Table IV**).

**Table IV:** Risk of basal cell carcinoma (BCC) in mastocytosis patients compared with mastocytosis-free comparators

Item	No (%)	Yes (%)	Total	Diagnosis before index date
Cases	1,872 (91.8)	168 (8.2)	2040	73
Comparators	9,706 (95.2)	487 (4.8)	10,193	210
OR (95% CI)	1.79 (1.49–2.14)			

No = No BCC. Yes = BCC

### Timing and risk of basal cell carcinoma diagnosis in relation to date of mastocytosis diagnosis

Through further analyses the timing and risk of BCC in relation to index date was assessed. Of the 168 BCCs, 95 were diagnosed after index date and 73 cases before. When including cases also diagnosed 1 year before index date, the number increased to 113, yielding an HR of 1.91 (95% CI 1.54–2.37) (Fig. 1, **Table V**).

**Table V:** Risk of basal cell carcinoma (BCC) after mastocytosis diagnosis based on 3 scenarios: (1) after date of mastocytosis diagnosis, (2) including a 1-year period before mastocytosis diagnosis, (3) including a 3-year prior period

Item	Events	Person years	HR (95% CI)	*p*-values
Scenario 1				
Cases	95	53.9	1.77 (1.40–2.23)	< 0.001
Comparators	277	278.1	1.00 (ref)	
Scenario 2				
Cases	113	59.3	1.91 (1.54–2.37)	< 0.001
Comparators	304	305.5	1.00 (ref)	
Scenario 3				
Cases	129	70.2	1.90 (1.55–2.33)	< 0.001
Comparators	348	360.4	1.00 (ref)	

## DISCUSSION

Earlier studies have reported an association between mastocytosis and MM (10, 12–19, 22), findings that have been hypothesized to reflect common pathogenesis including a potential role of growth-promoting cytokines produced by MCs and co-occurrence of KIT mutations.

Based on the largest population-based study to date, our results broadly corroborate earlier findings in that a diagnosis of MM/Mis was more common in individuals with mastocytosis compared with the background population represented by mastocytosis-free comparators.

However, our findings of a risk increase of both MM/Mis and BCC – skin tumours originating from different cell types, melanocytes, and keratinocytes, respectively, and without common pathogenesis – point to an influence of other factors. First, we found that a substantial proportion of MM/Mis and BCC cases were detected near the date of mastocytosis diagnosis, pointing to an influence of detection bias. Thus, skin tumours may have been detected during work-up to establish the mastocytosis diagnosis and regular skin check-ups post diagnosis. Second, UV exposure is a strong risk factor for both MM/Mis and BCC. Lifetime UV exposure is likely to be high in individuals with mastocytosis because of UV treatment and patients’ own sun exposure to alleviate symptoms such as pruritus. The role of UV treatment for dermatological conditions and the associated carcinogenetic risks have been documented previously (23, 24).

### Strengths and limitations

The main strengths of our study included the population-based setting, the size of the study, and the availability of a matched comparison cohort. In addition, data were retrieved from several nationwide registry sources covering both hospital in- and outpatient care, minimizing the risk of selection bias. Compared with previous studies (10), we did not exclude patients with previous cancers, hence a baseline prevalence was calculated and included in our results.

Limitations included the lack of information on WHO classification of mastocytosis subtypes in the registers that provided data. Thus, we were unable to assess whether the incidence of MM/Mis/NMSC differs between subtypes. Furthermore, due to a limited time of follow-up, the estimates of mastocytosis-related skin tumours may be underestimated, as mastocytosis patients are generally younger than the age for peak incidence of MMs and NMSC. The data at hand did not include information on factors that could influence the risk of developing MM/Mis and NMSC, such as the history of UV treatment and sun exposure. Finally, because no information was available on MM stage, apart from the separation of MM from Mis, we were unable to assess whether the stage distribution of MM differed between the mastocytosis patients and the background population.

Taken together, corroborating results from earlier reports we found an increased risk of MM/Mis and BCC in mastocytosis patients. In the present study, we also found that a substantial proportion of skin cancers were detected near the date of mastocytosis diagnosis, a finding likely to reflect at least in part an influence of detection bias. Further research is needed to improve the understanding of diagnostic and pathogenic factors influencing the risk of skin cancer in individuals with mastocytosis, including the role of UV exposure.

## Supplementary Material



## References

[1] Dahlin JS, Maurer M, Metcalfe DD, Pejler G, Sagi-Eisenberg R, Nilsson G. The ingenious mast cell: contemporary insights into mast cell behavior and function. Allergy 2022; 77: 83–99. 10.1111/all.14881 33955017

[2] Maurer M, Köberle M, Metz M, Biedermann T. Mast cells: promoters of health and modulators of disease. J Allergy Clin Immunol 2019; 144: S1–3. 10.1016/j.jaci.2019.01.048 30826362

[3] Valent P, Akin C, Hartmann K, Alvarez-Twose I, Brockow K, Hermine O, et al. Updated diagnostic criteria and classification of mast cell disorders: a consensus proposal. HemaSphere 2021; 5: e646. 10.1097/HS9.0000000000000646 34901755 PMC8659997

[4] Hartmann K, Escribano L, Grattan C, Brockow K, Carter MC, Alvarez-Twose I, et al. Cutaneous manifestations in patients with mastocytosis: consensus report of the European Competence Network on Mastocytosis; the American Academy of Allergy, Asthma & Immunology; and the European Academy of Allergology and Clinical Immunology. J Allergy Clin Immunol 2016; 137: 35–45. 10.1016/j.jaci.2015.08.034 26476479

[5] Aberer E, Sperr WR, Bretterklieber A, Avian A, Hadzijusufovic E, Kluin-Nelemans HC, et al. Clinical impact of skin lesions in mastocytosis: a multicenter study of the European Competence Network on Mastocytosis. J Invest Dermatol 2021; 141: 1719–1727. 10.1016/j.jid.2020.12.030 33581142

[6] Bergström A, Hägglund H, Berglund A, Nilsson G, Lambe M. Epidemiology of mastocytosis: a population-based study (Sweden). Acta Oncol 2024; 63: 44–50. 10.2340/1651-226X.2024.31406 38380845 PMC11332469

[7] Gülen T, Akin C. Anaphylaxis and mast cell disorders. Immunol Allergy Clin North Am 2022; 42: 45–63. 10.1016/j.iac.2021.09.007 34823750

[8] Donker ML, Bakker NA, Jaspers WJM, Verhage AH. Two patients with osteoporosis: initial presentation of systemic mastocytosis. J Bone Miner Metab 2008; 26: 199–202. 10.1007/s00774-007-0800-x 18301978

[9] Greene LW, Asadipooya K, Corradi PF, Akin C. Endocrine manifestations of systemic mastocytosis in bone. Rev Endocr Metab Disord 2016; 17: 419–431. 10.1007/s11154-016-9362-3 27239674

[10] Broesby-Olsen S, Farkas DK, Vestergaard H, Hermann AP, Møller MB, Mortz CG, et al. Risk of solid cancer, cardiovascular disease, anaphylaxis, osteoporosis and fractures in patients with systemic mastocytosis: a nationwide population-based study. Am J Hematol 2016; 91: 1069–1075. 10.1002/ajh.24490 27428296

[11] Kibsgaard L, Deleuran M, Flohr C, Langan S, Braae Olesen A, Vestergaard C. How “benign” is cutaneous mastocytosis? A Danish registry-based matched cohort study. Int J Womens Dermatol 2020; 6: 294–300. 10.1016/j.ijwd.2020.05.013 33015290 PMC7522902

[12] Todd P, Garioch J, Seywright M, Rademaker M, Thomson J. Malignant melanoma and systemic mastocytosis – a possible association? Clin Exp Dermatol 1991; 16: 455–457. 10.1111/j.1365-2230.1991.tb01235.x 1806323

[13] Kowalzic L, Eickenscheidt L, Seidel C, Kribus S, Ziegler H, Komar M. Telangiectasia macularis eruptiva perstans, a form of cutaneous mastocytosis, associated with malignant melanoma. J Dtsch Dermatol Ges 2009; 7: 360–362. 10.1111/j.1610-0387.2008.06941.x 19054427

[14] Hägglund H, Sander B, Gülen T, Lindelöf B, Nilsson G. Increased risk of malignant melanoma in patients with systemic mastocytosis? Acta Derm Venereol 2014; 94: 583–584. 10.2340/00015555-1788 24473924

[15] Donati P, Paolino G, Donati M, Panetta C. Cutaneous mastocytosis combined with eruptive melanocytic nevi and melanoma: coincidence or a linkage in the pathogenesis? J Dermatol Case Rep 2014; 8: 70–74. 10.3315/jdcr.2014.1179 25324908 PMC4195503

[16] Ruini C, Hartmann D, Flaig MJ, von Braunmühl T, Berking C. [Aggressive malignant melanoma in a patient with urticaria pigmentosa]. Hautarzt Z Dermatol Venerol Verwandte Geb 2018; 69: 45–48. 10.1007/s00105-018-4220-8 30264298

[17] Capo A, Goteri G, Mozzicafreddo G, Serresi S, Giacchetti A. Melanoma and mastocytosis: is really only a coincidence? Clin Exp Dermatol 2019; 44: 76–77. 10.1111/ced.13717 30178486

[18] Akdogan N, Elcin G, Gokoz O. The co-existence of cutaneous melanoma and urticaria pigmentosa in a patient with Becker’s nevus. J Cosmet Dermatol 2020; 19: 1268–1270. 10.1111/jocd.13243 31803997

[19] Rydberg A, Lehman J, Markovic S, Anderson K. Mastocytosis and melanoma: a case series. Int J Dermatol 2022; 61: 603–606. 10.1111/ijd.15791 34350975

[20] Svenska melanomregistret (SweMR) – kvalitetsregisterrapport, 1990–2022. https://cancercentrum.se/globalassests/om-rcc/sydost/pdf/nationell-kvalitetsregisterrapport-hudmelanom-1990-2022.pdf

[21] Kappelin J, Green AC, Ingvar Å, Ahnlide I, Nielsen K. Incidence and trends of basal cell carcinoma in Sweden: a population-based registry study. Br J Dermatol 2022; 186: 963–969. 10.1111/bjd.20964 34939666

[22] Vojvodic A, Vlaskovic-Jovicevic T, Vojvodic P, Vojvodic J, Goldust M, Peric-Hajzler Z, et al. Melanoma and mastocytosis. Open Access Maced J Med Sci 2019; 7: 3050–3052. 10.3889/oamjms.2019.772 31850121 PMC6910818

[23] Archier E, Devaux S, Castela E, Gallini A, Aubin F, Le Maître M, et al. Carcinogenic risks of psoralen UV-A therapy and narrowband UV-B therapy in chronic plaque psoriasis: a systematic literature review. J Eur Acad Dermatol Venereol 2012; 26: 22–31. 10.1111/j.1468-3083.2012.04520.x 22512677

[24] Stern RS. The risk of squamous cell and basal cell cancer associated with psoralen and ultraviolet A therapy: a 30-year prospective study. J Am Acad Dermatol 2012; 66: 553–562. 10.1016/j.jaad.2011.04.004 22264671

