# Supplementary material for: Evaluating Melanoma Risk in Adult Mastocytosis: Potential Impact of Detection Bias – A Registry-based Study (Sweden)
Source: Acta Derm Venereol. 2025 Aug 18;105:43052. doi: 10.2340/actadv.v105.43052 (PMC12371744; doi:10.2340/actadv.v105.43052)
Supplement: Supplementary file 1 [file ActaDV-105-43052-s1.pdf]

Supplementary material has been published as submitted. It has not been copyedited, or typeset by Acta Dermato-Venereologica

Supplementary

Table SI. ICD and SNOMED codes used to identify a cohort of mastocytosis patients

Diagnosis	ICD-10	SNOMED
Cutaneous mastocytosis	Q822	97401
- Urticaria pigmentosa	Q822A	
- DCM	Q822B	
- Mastocytoma	Q822C	
- TMEP	Q822D	
- Mastocytosis unspec	Q822X	
Systemic mastocytosis	Q822	97411, 97413
Mast cell leukemia	C943	97423
Malignant mast cell tumor, Aggressive SM	C962	97413
Mast cell tumor of unknown nature	D470	97411
Mast cell sarcoma	C962	97403
Malignant melanoma	C43	
Melanoma in situ	D03	
Basal cell carcinoma	C44.0-9B-E	

Table SII. Number of patients and diagnostic codes by register source

Diagnostic codes, NPR, outpatient

	n	%
C943	9	0.5
C962	61	3.1
D470	296	15.4
Q822	477	24.9
Q822A	804	42.0
Q822B	28	1.5
Q822C	56	2.9
Q822D	88	4.6
Q822X	123	6.4

Diagnostic codes, NPR, inpatient

	n	%
C943	3	0.1
C962	39	11.8
D470	71	21.5
Q822	108	32.7
Q822A	49	14.8
Q822B	31	9.3
Q822C	1	0.0
Q822D	3	0.1
Q822X	24	7.3

Diagnostic codes, SCR

	n	%
97401	19	10.8
97403	2	1.1
97411	101	57.7
97413	50	28.6
97423	3	1.7
